# Neuroprotective and Neuromodulatory Effects Induced by Cannabidiol and Cannabigerol in Rat Hypo-E22 cells and Isolated Hypothalamus

**DOI:** 10.3390/antiox9010071

**Published:** 2020-01-13

**Authors:** Viviana di Giacomo, Annalisa Chiavaroli, Giustino Orlando, Amelia Cataldi, Monica Rapino, Valentina Di Valerio, Sheila Leone, Luigi Brunetti, Luigi Menghini, Lucia Recinella, Claudio Ferrante

**Affiliations:** 1 Department of Pharmacy, Università degli Studi “Gabriele d’Annunzio”, via dei Vestini 31, 66100 Chieti, Italy; viviana.digiacomo@unich.it (V.d.G.); annalisa.chiavaroli@unich.it (A.C.); amelia.cataldi@unich.it (A.C.); sheila.leone@unich.it (S.L.); luigi.brunetti@unich.it (L.B.); luigi.menghini@unich.it (L.M.); lucia.recinella@unich.it (L.R.); claudio.ferrante@unich.it (C.F.); 2 Genetic Molecular Institute of CNR, Unit of Chieti, “G. d’ Annunzio” University, Via dei Vestini 31, 66100 Chieti-Pescara, Italy; m.rapino@unich.it; 3 Department of Medicine and Ageing Sciences, “G. d’ Annunzio” University, Via dei Vestini 31, 66100 Chieti-Pescara, Italy.; valentina.divalerio@unich.it

**Keywords:** cannabidiol, cannabigerol, hypothalamus, neurotransmitter, neuropeptide, kinurenine pathway

## Abstract

Background: Cannabidiol (CBD) and cannabigerol (CBG) are non-psychotropic terpenophenols isolated from *Cannabis sativa*, which, besides their anti-inflammatory/antioxidant effects, are able to inhibit, the first, and to stimulate, the second, the appetite although there are no studies elucidating their role in the hypothalamic appetite-regulating network. Consequently, the aim of the present research is to investigate the role of CBD and CBG in regulating hypothalamic neuromodulators. Comparative evaluations between oxidative stress and food intake-modulating mediators were also performed. Methods: Rat hypothalamic Hypo-E22 cells and isolated tissues were exposed to either CBD or CBG, and the gene expressions of neuropeptide (NP)Y, pro-opiomelanocortin (POMC) and fatty acid amide hydrolase were assessed. In parallel, the influence of CBD on the synthesis and release of dopamine (DA), norepinephrine (NE), and serotonin (5-HT) was evaluated. The 3-hydroxykinurenine/kinurenic acid (3-HK/KA) ratio was also determined. Results: Both CBD and CBG inhibited NPY and POMC gene expression and decreased the 3-HK/KA ratio in the hypothalamus. The same compounds also reduced hypothalamic NE synthesis and DA release, whereas the sole CBD inhibited 5-HT synthesis. Conclusion: The CBD modulates hypothalamic neuromodulators consistently with its anorexigenic role, whereas the CBG effect on the same mediators suggests alternative mechanisms, possibly involving peripheral pathways.

## 1. Introduction

*Cannabis sativa* has long been considered as an efficacious pharmacological tool to treat a wide plethora of diseases, including fever, malaria, constipation, menstrual disorders, pain, and rheumatism. The plant, belonging to the Cannabaceae family and classified in the three recognized varieties (*sativa*, *indica* and *ruderalis)*, is characterized by the presence of numerous terpenophenolic compounds that are responsible, at least partially, for the aforementioned effects. The main compounds present in the phyto-complex are Δ9-tetrahydrocannabinol (THC), which is also the sole psychotropic molecule, and cannabidiol (CBD), which is structurally related to the first but devoid of psychotropic activity [1]. In the last three decades, the study of the pharmacological properties of these phyto-compounds have led to the identification and characterization of the endocannabinoid signaling, consisting in arachinonic acid-deriving molecules including anandamide and 2-acylglycerole, and the related metabotropic receptors, namely, the cannabinoid type 1 (CB1), expressed in prevalence at the presynaptic level, and the CB2, which is present especially but not exclusively at the peripheral level [2]. The endocannabinoid-mediated activation of CB1 and CB2 has long been implicated in the onset of neuroprotective effects, whereas the neuroprotection occurring after phyto-cannabinoid administration could be better explained by a multitarget mechanism involving different receptor systems, including peroxisome proliferator-activated receptors (PPARs) and transient receptor potential (TRP) channels [3,4,5]. CB1 and CB2 receptors were considered as promising targets for the development of anti-obesity drugs [6] as well, to the point that the rimonabant, the prototype of the of CB1 blockers, was clinically employed for a short period (more than ten years ago) for its anorexigenic effects. Nevertheless, the rimonabant was soon after retired from the market for an increased frequency in psychiatric disorders following the treatment [7]. The therapeutic failure was ascribed, at least partially, to the specific mechanism of action of the drug, whose orthosteric inverse agonism on CB1 could lead to supra-physiological receptor alterations [8]. To this regard, the CBD could represent an innovative pharmacological approach: even though it is described as a CB1 ligand, the CBD was reported to have a low affinity for the CB1 orthosteric site [9], and this could be considered as one of the main factors influencing the lack of psychiatric symptoms following the administration in vivo [10]. Additionally, Laprairie and colleagues [11] suggested that the cannadidiol could act as a negative allosteric modulator rather than an orthosteric ligand. CB1 negative allosteric modulators are molecules devoid of intrinsic receptor activity, which depends solely on the presence of the endogenous ligands, i.e., the endocannabinoids [8,12]. Considering that the brain level of endocannabinoids is upregulated in obese mice [13], the CBD could reduce, without annulling, the endocannabinoid-stimulated CB1 signaling, thus restoring the physiological activity of the endocannabinoid system [11]. Another possible anti-obesity target is the CB2 receptor. Ignatowska-Jankowska and colleagues [14] demonstrated that the anorexigenic effect following CBD administration could depend on the activation of the hypothalamic CB2 pool. Therefore, the anorexigenic effect exerted by the CBD could be the result of a multitarget mechanism, involving the whole endocannabinoid receptor system, particularly in the hypothalamus. Nevertheless, the potential influence of the cannabidiol on the hypothalamic appetite-regulating network, including the involvement of neuropeptides and neurotransmitters long considered as central transducers of peripheral appetite and satiety signals [15], has not been studied up to now. Consequently, the aim of the present research is to investigate the role of the CBD in regulating the levels of the hypothalamic peptides and neurotransmitters involved in feeding control, isolated rat hypothalamus, and the hypothalamic Hypo-E22 cell line. Additionally, taking into consideration many studies reporting the appetite-stimulating effects of another terpenophenol present in the *C. sativa* phyto-complex, that is cannabigerol (CBG) [16,17], which was also demonstrated to act as a negative allosteric modulator of CB1, [11], an evaluation of CBG effects on the experimental paradigms was performed as well.

## 2. Materials and Methods

### 2.1. Drugs

The crystals of CBD and CBG (99% purity, 1% terpene fraction), derived from concentrated extracts of *C. sativa*, were kindly provided by Enecta B.V. (Corantijnstraat 5–1, 1058DA Amsterdam, Netherlands). The mother solutions (30 mM) were prepared in dimethylsulfoxide (DMSO) and sterilized with 0.22 µm Millipore filters in sterility conditions (laminar flow hood). Afterwards, drug solutions were stepwise diluted in Dulbecco’s modified Eagle’s medium (DMEM) for the bio-pharmacological assays, as described below.

### 2.2. In Vitro Studies

The Hypo-E22 rat hypothalamus cell line was purchased from Cedarlane Corporation (Burlington, ON, Canada) and cultured in DMEM supplemented with 10% (*v*/*v*) heat-inactivated fetal bovine serum and penicillin-streptomycin (100 μg/mL) (all from EuroClone SpA Life-Sciences-Division, Milano, Italy). Cells were grown at 37 °C in a humified atmosphere of 5% CO_2_. When indicated, the cells were treated with H_2_O_2_ 300 μM for 3 h and different concentrations of CBD and CBG (1, 10, 100, 1000 nM). The cell viability was evaluated after 24 and 48 h of culture by MTT (3-[4,5-dimethyl-thiazol-2-yl]-2,5-diphenyl tetrazolium bromide) growth assay (Sigma–Aldrich, St. Louis, MO, USA), based on the capability of viable cells to reduce MTT into a colored formazan product. The cells were seeded into 96-well plates at 5 × 10^3^ cells/well. At the established time points, the medium was replaced with a fresh one containing 0.5 mg/mL MTT, and the cells were incubated for 3 h at 37 °C. After a further incubation of the samples in DMSO for 30 min at 37 °C the absorbance at 570 nm was measured using a Multiscan GO microplate spectrophotometer (Thermo Fisher Scientific, Waltham, MA, USA). The values obtained in the absence of cells were considered as background and subtracted from the optical density values of the samples. Three independent experiments were performed under the same experimental conditions. For the HPLC analyses, Hypo-E22 cells were seeded in 6-well plates at 10^5^ cells/well. After 24 h of exposure to CBD 1000 nM and CBG 1 nM, 300 µL of medium for each experimental condition were collected and stored at −20°C.

### 2.3. Ex Vivo Studies

Twenty-four male adult Sprague–Dawley rats (200–250 g) were housed in Plexiglass cages (40 × 25 × 15 cm), two rats per cage, in climatized colony rooms (22 ± 1 °C; 60% humidity), on a 12 h/12 h light/dark cycle (light phase: 07:00–19:00 h), with free access to tap water and food, 24 h/day throughout the study, with no fasting periods. Rats were fed a standard laboratory diet (3.5% fat, 63% carbohydrate, 14% protein, 19.5% other components without caloric value; 13.39 kJ/g). Housing conditions and experimentation procedures were strictly in accordance with the European Union ethical regulations on the care of animals for scientific research. The experimental paradigm was approved by the Local Ethical Committee (University “G. d’Annunzio” of Chieti-Pescara, Chieti-Pescara, Italy) and the Italian Health Ministry (Authorization N. F4738.N.XTQ, delivered on 11 Novembre 2018). Specifically, rats were sacrificed by CO_2_ inhalation (100% CO_2_ at a flow rate of 20% of the chamber volume per min) and hypothalami were immediately collected and maintained in humidified incubator with 5% CO_2_ at 37 °C for 4 h (incubation period), in DMEM enriched with CBD (1000 nM) or CBG (1 nM). Afterwards, the hypothalamic samples were subjected to analytical procedure for gene expression and biogenic amine level assessment, as described in the following paragraphs. The samples (*n* = 12) for the determination of biogenic amines were dissected in 1 mL of a perchloric acid solution (50 mM) and filtered (PTFE 0.45 µm), whereas the hypothalami (*n* = 12) intended to be used for gene expression analysis were dissected and stored in RNAlater solution (Ambion, Austin, TX, USA) at −20 °C.

### 2.4. RNA Extraction, Reverse Transcription and Real-Time Reverse Transcription Polymerase Chain Reaction (Real-Time RT PCR)

Total RNA was extracted from the hypothalamus using TRI Reagent (Sigma–Aldrich, St. Louis, MO, USA), as previously reported [18]. Contaminating DNA was removed using 2 units of RNase-free DNase 1 (DNA-free kit, Ambion, Austin, TX, USA). The RNA concentration was quantified at 260 nm by spectrophotometer reading (BioPhotometer, Eppendorf, Hamburg, Germany) and its purity was assessed by the ratio at 260 and 280 nm readings. The quality of the extracted RNA samples was also determined by electrophoresis through agarose gels and staining with ethidium bromide, under UV light. One microgram of total RNA extracted from each sample in a 20 μL reaction volume was reverse transcribed using a High Capacity cDNA Reverse Transcription Kit (Thermo Fisher Scientific Inc., Monza, Italy). Reactions were incubated in a 2720 Thermal Cycler (Thermo Fisher Scientific Inc., Monza, Italy) initially at 25 °C for 10 min, then at 37 °C for 120 min, and finally at 85 °C for 5 s. Gene expression was determined by quantitative real-time PCR using TaqMan probe-based chemistry. PCR primers and TaqMan probes, including β-actin used as the housekeeping gene, were purchased from Thermo Fisher Scientific Inc. (Assays-on-Demand Gene Expression Products, Rn00595020_m1 for pro-opiomelanocortin (POMC) gene, Rn00561681_m1 for neuropeptide (NP)Y gene, Rn00577086_m1 for fatty acide amide hydrolase (FAAH) gene). The real-time PCR was carried out in triplicate for each cDNA sample in relation to each of the investigated genes. Data were elaborated with the Sequence Detection System (SDS) software version 2.3 (Thermo Fisher Scientific Inc.). Gene expression was relatively quantified by the comparative 2^−ΔΔCt^ method [19].

### 2.5. High Performance Liquid Chromatography (HPLC) Determination of Dopamine (DA), Norepinephrine (NE),Serotonin (5-HT), and 3-Hydroxykinurenine (3-HK)

Tissue and extracellular DA, 5-HT and NE levels were analyzed through an HPLC apparatus consisting of a Jasco (Tokyo, Japan) PU-2080 chromatographic pump and an ESA (Chelmsford, MA, USA) Coulochem III coulometric detector, equipped with a microdialysis cell (ESA-5014b) porous graphite working electrode and a solid state palladium reference electrode. The analytical conditions for biogenic amine identification and quantification were selected according to a previous study [20]. Briefly, the analytical cell was set at −0.150 V for detector 1 and at +0.300 V for detector 2, with a range of 100 nA. The chromatograms were monitored at the analytical detector 2. Integration was performed by Jasco Borwin Chromatography software version 1.5. The chromatographic separation was performed by isocratic elution on a Phenomenex Kinetex reverse phase column (C18, 150 × 4.6 mm i.d., 2.6 µm). As regards the separation of DA, NE and 5-HT, the mobile phase was (10:90, *v/v*) acetonitrile and 75 mM pH 3.00 phosphate buffer containing octanesulfonic acid 1.8 mM, EDTA 30 µM and triethylamine 0.015% *v/v*. The mobile phase for 3-HK analysis consisted of 1.5% acetonitrile, 0.9% triethylamine, 0.59% phosphoric acid, 0.27 mM EDTA, and 8.9 mM octanesulfonic acid. Flow rate was 0.6 mL/min and the samples were manually injected through a 20 µl loop. Neurotransmitter peaks were identified by comparison with the retention time of pure standard. Neurotransmitter concentrations in the samples were calculated by linear regression curve (*y* = b*x* + m) obtained with standard. Neither internal nor external standard were necessary for neurotransmitter quantification in the hypothalamus homogenate, and all tests performed for method validation yielded results in accordance with limits indicated in official guidelines for applicability in laboratory trials. The standard stock solutions of DA, NE, and 5-HT at 2 mg/mL were prepared in bidistilled water containing 0.004% EDTA and 0.010% sodium bisulfite. The stock solutions were stored at 4 °C. Work solutions (1.25–20.00 ng/mL) were obtained daily by progressively diluting the stock solutions in the mobile phase.

### 2.6. HPLC-Fluorimetric Determination of Kinurenic Acid (KA)

The KA quantitative determination in the cell medium was carried out on a reversed phase HPLC-fluorimeter in agreement with the method employed by Pocivavsek and colleagues [21]. Analyses were performed by using a liquid chromatograph (MOD. 1525, Waters Corporation, Milford, MA, USA) equipped with a fluorimetric detector (MOD. 2475, Waters Corporation), a C18 reversed-phase column (AcclaimTM 120, 3 µm, 2.1 × 100 mm, Dionex Corporation, Sunnyvale, CA, USA), and an on-line degasser (Biotech 4-CH degasi compact, LabService, Anzola Emilia, Italy). The separation was conducted in isocratic conditions and the mobile phase consisted of 250 mM zinc acetate, 50 mM sodium acetate, and 3% acetonitrile (pH adjusted to 6.2 with glacial acetic acid), using a flow rate of 1.0 mL/min. In the eluate, the KA was identified and measured fluorimetrically (excitation: 344 nm; emission: 398 nm).

### 2.7. Statistical Analysis

Statistical analysis was carried out through GraphPad Prism version 5.01 for Windows (GraphPad Software, San Diego, CA, USA). Means ± S.D. were determined for each experimental group and analyzed by one-way analysis of variance (ANOVA), followed by Newman–Keuls comparison multiple test. Statistical significance was set at *p* < 0.05. As regards the animals employed for the experiments, their number per condition (*n* = 4) was calculated with the software G*Power (v3.1.9.4, UCLA, Los Angeles, CA, USA). The values of study potency (1-β) and significance level (α) were 0.8 and 0.05, respectively.

## 3. Results

**Figure 1:**
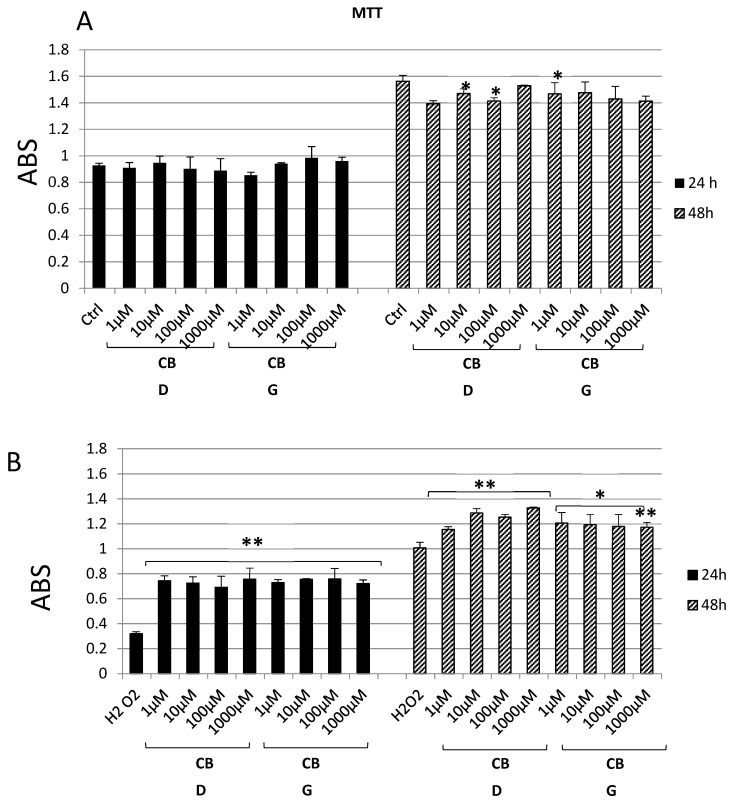
MTT assay of hypothalamic Hypo-E22 cells exposed to different concentrations (1–1000 nM) of either cannabidiol (CBD) or cannabigerol (CBG) for 24 and 48 h. (**A**) Cells in basal conditions. (**B**) Cells challenged with 300 µM H_2_O_2_. ANOVA, *p* < 0.001; ** *p* < 0.01, * *p* < 0.05 vs. control group.

Cannabidiol and cannabigerol show no significant effect on Hypo-E22 proliferation when they are added to the cell medium at different concentrations for 24 h (Figure 1A). On the contrary, the MTT assay at 48 h shows that, whereas the CBD 1000 nM has an OD value similar to the control, the lower CBD concentrations (1, 10, 100 nM) and all the CBG concentrations appear to induce a slight decrease in cell viability, even though it is not always significant. On the other hand, when the hypothalamic cell line is challenged with hydrogen peroxide (Figure 1B), its viability is markedly reduced (0.32 ± 0.01 H_2_O_2_ vs. 0.93 ± 0.06 ctrl 24 h and 1.00 ± 0.04 H_2_O_2_ vs. 1.56 ± 0.05 ctrl 48 h). The addition of the two substances to the cell culture results in an improved viability for all the experimental points. Both after 24 and 48 h of exposure, CBD and CBG are able to protect the cells from the oxidative stress induced by the hydrogen peroxide. For the subsequent analyses, two concentrations were chosen, namely, 1000 nM for CBD, having shown the best recover from the H_2_O_2_ damage at both 24 h (0.76 ± 0.10 vs. 0.32 ± 0.01 of the H_2_O_2_ sample) and 48 h (1.32 ± 0.01 vs. 1.00 ± 0.04 of the H_2_O_2_ sample) and 1 nM for CBG, being the lowest concentration to give the highest cell viability (0.73 ± 0.02 at 24 h and 1.21 ± 0.08 at 48 h).

**Figure 2:**
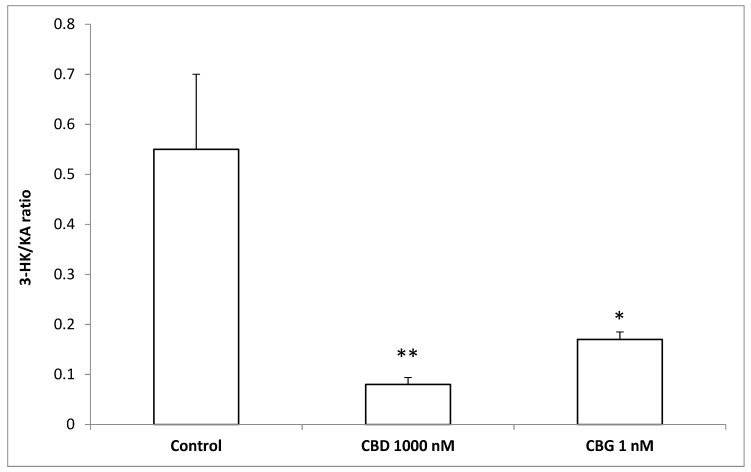
Inhibitory effects induced by cannabidiol (CBD) 1000 nM and cannabigerol (CBG) 1 nM on extracellular 3-hydroxykinurenine/kynurenic acid (3-HK/KA) ratio in hypothalamic Hypo-E22 cells. ANOVA, *p* < 0.001; ** *p* < 0.01, * *p* < 0.05 vs. control group.

**Figure 3:**
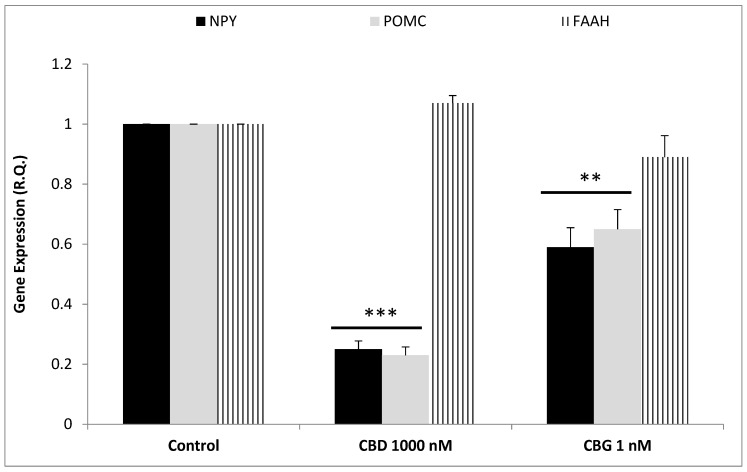
Inhibitory effects induced by cannabidiol (CBD) 1000 nM and cannabigerol (CBG) 1 nM on neuropeptide (NP)Y and pro-opimelanocortin (POMC) gene expression (Relative Quantification: R.Q.), in isolated rat hypothalamus. ANOVA, *p* < 0.0001; *** *p* < 0.001, ** *p* < 0.01 vs. control group. Conversely, CBD and CBG exerted a null effect on fatty acid amide hydrolase (FAAH) gene expression.

**Figure 4:**
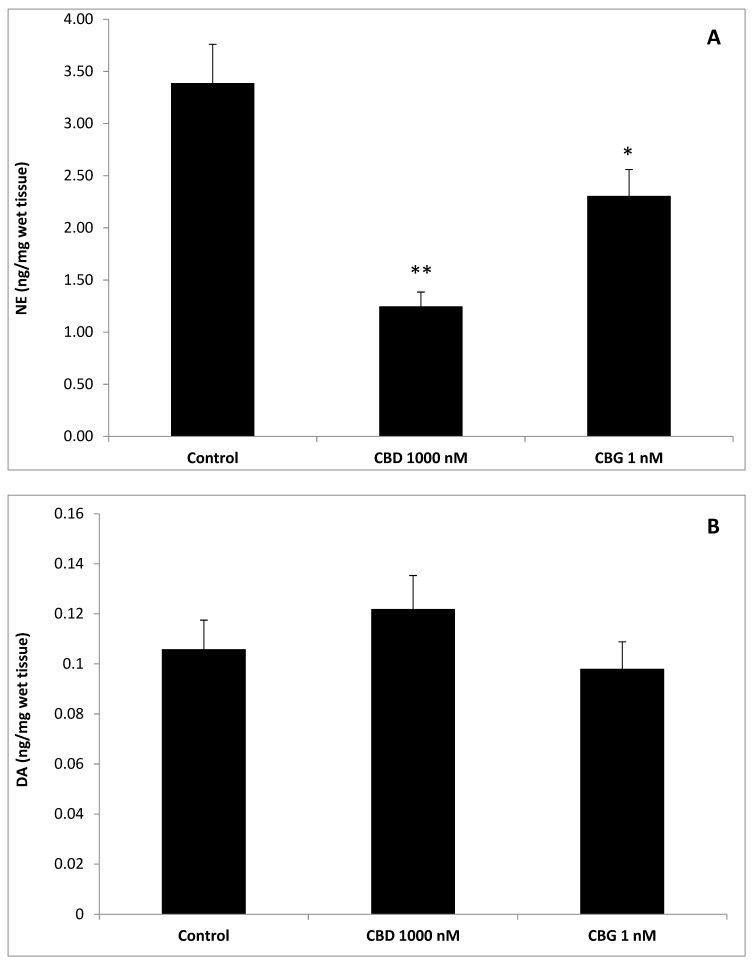

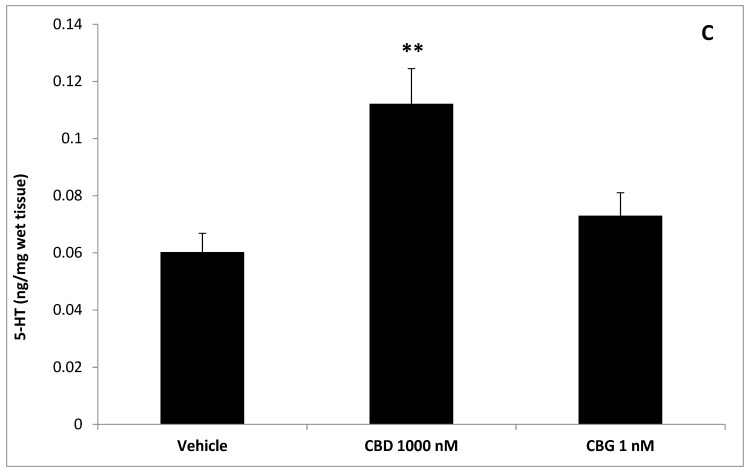
Effects of cannabidiol (CBD) 1000 nM and cannabigerol (CBG) 1 nM on norepinephrine (NE), dopamine (DA), and serotonin (5-HT) levels (ng/mg wet tissue) in isolated rat hypothalamus. (**A**) CBD and CBG inhibited NE level in the hypothalamus. ANOVA, *p* < 0.0001; *** *p* < 0.001, ** *p* < 0.01 vs. control group. (**B**) Neither CBD nor CBG influenced DA level, in the hypothalamus. (**C**) Conversely, CBD and CBG stimulated hypothalamic 5-HT level. ANOVA, *p* < 0.001; ** *p* < 0.01 vs. control group.

**Figure 5:**
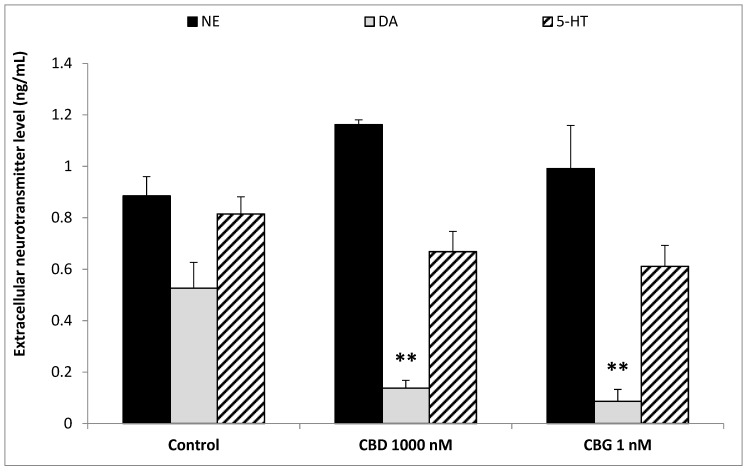
Inhibitory effects induced by cannabidiol (CBD) 1000 nM and cannabigerol (CBG) 1 nM on extracellular dopamine (DA) levels (ng/mL), in hypothalamic Hypo-E22 cells. ANOVA, *p* < 0.001; ** *p* < 0.01 vs. control group. Conversely, CBD and CBG exerted a null effect on extracellular norepinephrine (NE) and serotonin (5-HT) levels.

To better evaluate the effect of cannibidiol and cannabigerol on the Hypo-E22 cells in their basal state, the detection of the extracellular release of 3-HK and KA was performed. The ratio 3-HK/KA, a well-known index of neurotoxicity, was considerably reduced following CBD and CBG treatment (Figure 2), with a higher efficacy found when the cells were exposed to CBD (0.08 ± 0.014 vs. 0.17 ± 0.015 of the CBG group). The stimulation of isolated rat hypothalamus with CBD 1000 nM and CBG 1 nM led to significant alterations in the expression pattern of the genes NPY and POMC, with a significant reduction (*p* < 0.0001) of their mRNA level (Figure 3) compared to control-treated group. On the other hand, both compounds revealed ineffective in altering the gene expression of FAAH (Figure 3). In parallel, a significant inhibition (*p* < 0.0001) of NE steady state level was observed (Figure 4A) when the hypothalami were exposed both to CBD and CBG. Conversely, a null effect was observed on DA concentration (Figure 4B), whereas only CBD proved able to increase the hypothalamic level of 5-HT, following the 4 h treatment (Figure 4C; *p* < 0.001). As for the effects of the two compounds on the modulation of the biogenic amine release from Hypo-E22 cells, both CBD and CBG decreased the DA level (*p* < 0.001) without affecting NE and 5-HT extracellular concentrations (Figure 5).

## 4. Discussion

The protective effects exerted by the two metabolites of *C. sativa* (CBD and CBG) on hypothalamic cells challenged with H_2_O_2_, as demonstrated by the MTT assay results, are confirmed by the modulation of the release of kynurenine metabolites by the same cells. The 3-HK and KA are key products of the kynurenine pathway, which represents, together with the 5-HT pathway, the two main tryptophan degradative systems [22]. Additionally, tissue and plasma levels of these two molecules are well known to be related to inflammatory and oxidative stress conditions in both peripheral and central tissues [23,24,25]. Specifically, the 3-HK/KA is a reliable marker of neurotoxicity [26], and our findings of reduction of this ratio from Hypo-E22 cells after pharmacological treatment further support the neuroprotective role exerted by both CBD and CBG. Feeding behavior and energy balance are finely modulated in the hypothalamus, which has long been considered a cornerstone in this process [27]. In this region of the brain, neuropeptides, including NPY and POMC, and biogenic amines, namely DA, NE, and 5-HT, act as central transducers of peripheral short- and long-term satiety signals [15]. Although the role of NPY as central appetite stimulant is well-established [15], the involvement of POMC is still controversial, having this neuropeptide demonstrated both stimulatory and inhibitory effects, depending on the post-transcriptional pathway activation, that could lead to the production of both the anorexigenic α-melanocyte stimulating hormone (α-MSH) and the orexigenic β-endorphin (β-END) [28]. In this regard, Koch and colleagues [29] suggested that cannabinoid-induced feeding could be mediated by increased levels of the POMC-derived peptide β-endorphin. Actually, the inhibition of the POMC gene expression following CBD and CBG treatment is consistent with their putative role as negative allosteric modulators of CB1 receptor [30]. In contrast, the inhibition of the expression of the NPY gene registered after the treatment, although being consistent with the anorexigenic effects ascribed to CBD [6], appears to be discrepant with the orexigenic role of CBG [16,17]. This result is, however, in agreement with the expression of the CB1 receptor on synaptic endings innervating both NPY and POMC first order neurons in the hypothalamus [29,31]. Interestingly, the anorexigenic effects induced by bisphenol A in mice were followed by the concomitant reduction and stimulation of CB1 and cocaine and amphetamine-regulating transcript (CART) peptide gene expression, respectively; thus, further highlighting the importance of hypothalamic arcuate nucleus first order neurons as key targets of the anti-obesity effects of CB1-modulating compounds [32]. Nevertheless, the study performed by Merroun and colleagues [33] suggested the lateral hypothalamus-derived orexin A as a mediator of the anorexigenic effects induced by CB1 antagonist AM251 as well [33]. The anorexigenic effects of CBD were also related to CB2 receptor activation [14], whilst the cannabigerol proved to challenge brain α2-adrenoceptor [34], whose activation is well known to be related to a feeding stimulating effect [35]. Another parameter investigated after CBD and CBG treatment was the gene expression of FAAH, a key enzyme known for being able to stimulate food intake and notoriously involved in the degradation of endocannabinoids such as anandamide [7]. The null effect on FAAH gene expression registered after the exposure to CBD and CBG is consistent with their low potency as FAAH inhibitors [36], thus excluding, in this experimental system, the direct involvement of endocannabinoid levels in mediating the observed modulatory effects in the hypothalamic appetite-regulating network. The steady state levels of DA, NE, and 5-HT in isolated hypothalamus, were assayed after the challenging with CBD and CBG. The role of DA on neuroendocrine control of food intake is still a matter of debate, being dependent on the hypothalamic site of administration for the result in stimulation or inhibition [37]. However, mesolimbic dopaminergic pathways seem to be involved in the reward underlying the ingestion of palatable foods, thus suggesting a stimulating effect on appetite [38]. Hypothalamic NE is involved in feeding regulation as well, with a role in inhibiting or stimulating the food intake mediated by α1- or α2-adrenoceptors, respectively, depending on the circadian alteration in the α1/α2 ratio [35]. Although multiple studies suggest the inhibition of the monoamine release as a possible mechanism of action of peripheral anorexigenic hormones [18,39,40], central 5-HT is well known to reduce appetite and increase energy expenditure [41], while its release in the hypothalamus could be increased by peripheral anorexigenic hormones [42]. Having both CBD and CBG demonstrated their effectiveness in reducing NE steady state level in isolated rat hypothalamus, this monoamine can be suggested as a possible mediator of the effects of the two terpenophenols on food intake. In a previous study, the RVD-hemopressin-α, an endogenous anorexigenic peptide, proved to be a negative allosteric modulator of CB1 [43] and to inhibit hypothalamic NE levels following peripheral administration despite being ineffective against DA and 5-HT levels [30]. The two terpenophenols objects of this study were also ineffective against the DA level, whereas the sole CBD stimulated 5-HT levels, and this could explain, albeit partially, the aforementioned anorexigenic effects [14]. The evaluation of the biogenic amine steady state level is currently considered a useful tool to predict the effect of a drug on the activity in the brain, particularly in in vivo studies [44,45]. Nevertheless, other experimental paradigms, such as microdialysis/push-pull, synaptosome perfusion, and cell cultures can give a more detailed assessment with regard to the effects of drugs on brain neurotransmitter release. In order to better elucidate a possible direct effect on aminergic signaling, the hypothalamic cell line Hypo-E22 was exposed to CBD and CBG to evaluate their influence on DA, NE, and 5-HT release. Since both CBD and CBG were able to reduce extracellular DA level in the cell line while there was no change in isolated rat hypothalamus, an inhibitory effect on neurotransmitter release, possibly from the ready-releasable vesicles, can be hypothesized. Conversely, the null effect of CBD on 5-HT release in the Hypo-E22 indicates that the increased 5-HT level in isolated hypothalamus, after CBD treatment, could happen through a stimulating effect on neurotransmitter synthesis. It should also be highlighted that the hypothalamic 5-HT level tended to increase, without resulting in significant, after CBG exposure. Intriguingly, both stimulating effects on 5-HT level were paralleled by a significant decrease in the 3-HK/KA ratio. Considering that pro-inflammatory conditions could activate the kinurenine pathway, thus leading to increased 3-HK/KA and 5-HT turnover [46], the inverse trend observed in the rat hypothalamus following CBD and CBG treatment suggests tryptophan degradative pathways as potential targets underlying the pharmacological effects of these compounds. In order to validate this hypothesis, a deepening further study is required to investigate the effects of CBD and CBG on kinurenine and tryptophan levels and the expression of the enzymes involved in the respective biochemical pathways.

## 5. Conclusions

Collectively, the present results indicate a modulation induced by both CBD and CBG on the hypothalamic neuropeptides and neurotransmitters playing a master role in feeding behavior. The increased 5-HT level, the reduced NPY and POMC gene expression, the NE tissue level, and the DA release are consistent with the anorexigenic role of CBD, after peripheral administration [14]. The CBG, on the contrary, besides being ineffective in modulating the hypothalamic 5-HT level, showed a pharmacological profile against the other tested neuromodulators that is very close to that of CBD. In this context, the involvement of other mechanisms and mediators not included in the present research could also explain the observed effects of CBG on orexigenic molecule balance following peripheral administration. Therefore, further investigations are needed to elucidate the effects of these compounds in the neuroendocrine mechanisms of feeding behavior, possibly taking into consideration the involvement of peripheral signals as well.
